# Multidrug Resistance among Patients with Surgical Site Infection in a Tertiary Care Centre

**DOI:** 10.31729/jnma.8353

**Published:** 2023-11-30

**Authors:** Anjan Palikhey, Subash Chandra Chaudhary, Santosh Mishra, Jharana Shrestha, Paribesh Gyawali

**Affiliations:** 1 Department of Pharmacology, Universal College of Medical Sciences, Bhairahawa, Rupandehi, Nepal; 2 Department of Pharmacy, Universal Colleae of Medical Sciences, Bhairahawa, Rupandehi, Nepal; 3 Department of Surgery, Universal Colleae of Medical Sciences, Bhairahawa, Rupandehi, Nepal; 4 Department of Biochemistry, Universal College of Medical Sciences, Bhairahawa, Rupandehi, Nepal; 5 Basantapur Primary Health Center, Basantapur, Rupandehi, Nepal

**Keywords:** *antimicrobial drug resistance*, *multidrug resistance*, *Staphylococcus aureus*, *surgical site infection*

## Abstract

**Introduction:**

Infections at surgical sites are a major problem all over the world. Pathogens linked to postoperative infections are becoming increasingly resistant to antibiotics, which presents a significant therapeutic challenge for surgeons and raises the financial burden placed on patients. The study aimed to find the prevalence of multidrug resistance among patients with surgical site infections in a tertiary care centre.

**Methods:**

A descriptive cross-sectional study was conducted from 3 November 2022 to 2 May 2023 among post-operative patients with surgical site infection after receiving ethical approval from the Institutional Review Committee. Swab samples were sent for antimicrobial susceptibility testing. Convenience sampling method was used. The point estimate was calculated at a 95% Confidence Interval.

**Results:**

Among 147 patients with surgical site infection, the prevalence of multi-drug resistance was 95 (64.63%) (56.90-72.36, 95% Confidence Interval). Among them, 51 (53.68%) patients were in the 21-40 years age group and most of them were male 53 (55.79%).

**Conclusions:**

The prevalence of multidrug-resistant cases was similar to other studies done in similar settings.

## INTRODUCTION

Surgical site infection (SSI) is the infection of the skin, hypodermic tissue, or adjacent organs/spaces caused by an operation and occurring within 30 days of the surgery.^1^ SSI is a common surgical complication. Many SSI-causing organisms are carried by the patient's own endogenous flora like *Staphylococcus aureus, Enterococcus, and Escherichia coli.^2^*

Antimicrobial drug resistance (AMR) has emerged as a major public health problem and is posing significant difficulties in the management of patients with SSIs. There has been a dramatic rise in AMR in Nepal over the past 20 years, with the prevalence of multidrug-resistant organisms also on the rise.^3^ In Nepal, the studies showing information on pyogenic microorganisms, such as their drug susceptibility pattern, are inconsistent.^4^ A thorough understanding of the microorganisms involved, their resistant nature, and their recently updated antimicrobial therapy plays a crucial role in the management.

The study aimed to find the prevalence of multidrug resistance among patients with surgical site infections in a tertiary care centre.

## METHODS

This was a descriptive cross-sectional study conducted at the post-operative ward of the Department of Surgery and Department of Gynaecology and Obstetrics of Universal College of Medical Sciences, Bhairahawa, Rupandehi, Nepal from 3 November 2022 to 2 May 2023, after obtaining ethical approval from Institutional Review Committee of the same institute (Reference number: UCMS/IRC/147/22). The patients of either sex or age above 18 years having positive swab/pus culture were included. The patients with suture abscesses, local puncture wounds, trauma-related wounds, episiotomy or infected burn wounds were excluded from the study. Convenience sampling method was used. The sample size of the study was calculated using the formula:


$$\begin{array}{ll} \text{n} & ={\text{Z}}^{2}\times \frac{\text{p}\times \text{q}}{{\text{e}}^{\text{2}}}\\ & =1.96^{2}\times \frac{0.50\times 0.50}{0.09^{2}}\\ & =119 \end{array}$$

Where,

n = minimum required sample size
Z = 1.96 at 95% Confidence Interval (CI)
p = prevalence taken as 50% for maximum sample calculation
q = 1-p
e = margin of error, 9%

The calculated sample size was 119. However, 147 patients were included in the study.

For the post-operative patient with surgical site infection occurring within 30 days after the operative procedure, swab/pus samples were sent for culture and antimicrobial susceptibility test (AST) to the microbiology department. The AST report data was collected from the patient's record, which includes the patient's laboratory report of pus culture results and antibiogram. The case pro forma was used as a tool for recording the patient's demographic information and AST report data, which was taken only after verbal and written consent from the patients. Samples were said to be multi-drug resistant (MDR) if bacteria showed resistance to at least one antibiotic from three or more relevant antibiotic groups.^5^

Data were entered and analysis was performed using IBM SPSS Statistics version 20.0. The point estimate was calculated at a 95% CI.

## RESULTS

Among 147 patients with SSIs, the prevalence of MDR was 95 (64.63%) (56.90-72.36, 95% Confidence Interval). The highest percentage of MDR was shown by *Staphylococcus aureus* 36 (37.89%) followed by *Escherichia coli* 20 (21.05%) and *Acinetobacter* Species 15 (15.79%) (Table 1).

**Table 1:** Multidrug resistance among bacteria causing SSIs (n= 95).

Bacteria	n (%)
**Gram-negative**	
*Escherichia coli*	20 (21.05)
*Acinetobacter species*	15 (15.79)
*Klebsiella pneumonia*	8 (8.42)
*Pseudomonas aeruginosa*	11(11.58)
**Gram-positive**	
*Staphylococcus aureus*	36 (37.89)
*Coagulase Negative*	5 (5.26)
*Staphylococcus aureus*	

Among MDR *Escherichia coli,* resistant to cefixime, levofloxacin, azithromycin was seen in 31 (77.50%), 16 (40%), 12 (30%). Among MDR *Acinetobacter,* resistance to cefixime, amoxyclav, and ceftriaxone was seen in 17 (89.47%), 12 (63.16%), and 11 (57.89%) respectively. Among MDR *Klebsiella pneumoniae*, resistant to cefixime, amoxyclav, and levofloxacin was seen in 11 (57.89%), 9 (47.37%), and 7 (36.84%) respectively. A total of 51 (53.68%) patients were in the 21-40 years age group and 53 (55.79%) were male (Table 2).

**Table 2:** Socio-demographic parameters (n= 95).

Variables	n (%)
**Age group (years)**	
<20	6 (6.32)
21-40	51 (53.68)
41-60	22 (23.16)
>60	16 (16.84)
**Gender**	
Male	53 (55.79)
Female	42 (44.21)

## DISCUSSION

The prevalence of multi-drug resistance among bacteria-causing surgical site infections was 95 (64.63%). In a previous study, gram-negative isolates 66.90% were found to MDR and gram-positive isolates 56.52% were found to MDR.^6^ In contrast to these findings, gram-positive bacteria were nearly 100% MDR and gram-negative were 95.50% MDR.^7^ Selfmedication, a dearth of diagnostic laboratory facilities, and a lack of guidelines for drug selection may all contribute to the widespread emergence of antibiotic-resistant strains in clinical isolates.

Gram-negative bacterial growth (60.55%) exceeded that of Gram-positive bacterial growth (39.45%) among the total 147 isolated species. Similar results were found in a 2015 study, which found that gram-negative bacteria accounted for 65.68% of the samples and gram-positive bacteria for 34.32%.^8^ In a previous study 27% of the bacteria were Gram-positive, whereas 73% were Gram-negative.^9^ Possible explanations for these discrepancies include differences in the research population, surgical location, and hospital setting.^10^ In our study, Staphylococcus aureus was the most common causative agent of SSIs 36.05% were consistent with those of previous studies.^11^ In our study, MDR due to gram-negative 56.80% bacteria isolated from surgical site infection were high, similar to the previous study.^12^

Common bacterial isolates like *Escherichia coli* (60%) and *Staphylococcus aureus* (66.66%) exhibited multidrug resistance to commonly used antibiotics like cefixime, azithromycin, levofloxacin, which is a matter of concern. On the contrary, in the previous study done among gram-negative bacterial isolates, *Proteus* species (91.6%) were found to have a higher frequency of MDR, whereas 81% of *Staphylococcus aureus* were reported as MDR.^12^ Most commonly used antibiotics were resistant in the present study as our research participants comprised indoor patients who developed infections from already resistant bacteria.

It is a single-centre study, hence the result could not be generalized to a larger setting.

## CONCLUSIONS

The prevalence of multidrug-resistant cases among patients with SSIs was similar to the other studies done in similar settings. Awareness regarding antibiotic resistance and antibiotic sensitivity tests in the laboratory should be mandatory before prescription to prevent antibiotic resistance.
